# Evolution of therapy for limited stage diffuse large B-cell lymphoma

**DOI:** 10.1038/s41408-021-00596-z

**Published:** 2022-02-24

**Authors:** Alexandra E. Rojek, Sonali M. Smith

**Affiliations:** 1 Department of Medicine, University of Chicago, Chicago, IL USA; 2 Section of Hematology/Oncology, Department of Medicine, University of Chicago, Chicago, IL USA

**Keywords:** Cancer immunotherapy, Radiotherapy

## Abstract

Diffuse large B-cell lymphoma (DLBCL) is the most common non-Hodgkin lymphoma (NHL), with limited-stage DLBCL defined as stage I or II disease. Risk stratification, initial treatment options, and relapse patterns are distinct from advanced-stage DLBCL, but there is limited data on the impact of biologic features on outcome. Patients have excellent outcomes, with ~90% survival at 2 years. Over the past several years, sequential prospective trials and large registry studies have evaluated the optimal number of chemotherapy cycles and implemented PET-adapted approaches to reduce the need for radiotherapy. Special consideration must still be given to cases of bulky disease, extranodal disease, fully resected scenarios, and adverse biologic features such as high-grade B-cell lymphoma with double/triple hit rearrangements. This review presents the evolution of a modern management approach, with a discussion of recent treatment-defining studies.

## Introduction and historical perspective

Diffuse large B-cell lymphoma (DLBCL) is the most common non-Hodgkin lymphoma (NHL), representing almost one-quarter of all new NHL cases per year in the United States [1]. It is biologically heterogeneous; historically, limited-stage disease was defined as Ann Arbor stage I or II disease with sites that could be encompassed in a single radiation field. Currently, limited or early-stage disease is defined as stage I or II, and advanced-stage disease is defined as stage III or IV by Lugano criteria [2]. Bulky disease, which is variably defined in the literature, is an important modifier to classical staging and is discussed further below. Large descriptive studies report that the median age of patients with the limited-stage disease is in the sixth decade of life, with a slight male predominance; [3–6], the most common anatomic sites of disease are in cervical lymph nodes and/or head and neck region, including Waldeyer’s ring. There remains debate as to whether the delineation between limited and advanced stage reflects earlier identification of a disease or a biologically distinct entity with different risks and outcomes features.

The modern approach to the treatment of limited-stage DLBCL was influenced by SWOG 8736. This phase 3 trial, conducted in the pre-rituximab era (1988–1995), established combined modality therapy as a standard of care in the pre-rituximab era. SWOG S8736 showed that three cycles of cyclophosphamide, doxorubicin, vincristine, and prednisone (CHOP) with radiation therapy (RT) were non-inferior to eight cycles of CHOP alone. This defined a new standard treatment length and inclusion of radiation for limited-stage patients [7] which has continued to serve as the foundation for subsequent dedicated limited-stage disease trials.

SWOG S8736 also developed a risk stratification scoring system in limited-stage DLBCL that retains utility in the modern era. The International Prognostic Index (IPI) for DLBCL was adjusted to better stratify prognosis in limited-stage disease by removing the number of extranodal sites and dichotomizing stage as I versus II. This stage-modified IPI (smIPI) thus includes one point each for age > 60 years, stage II disease, elevated serum lactate dehydrogenase (LDH), and performance status of two or above [7, 8]. In the pre-rituximab era, those with no risk factors, and thus stage I disease, had the best outcomes with 5-year overall survival (OS) of 95%; for those with one to two risk factors, 5-year OS dropped to 77%, and 50% for three or more risk factors [7, 9]. This smIPI model was more powerful than an age-adjusted risk stratification alone [10]. In the rituximab era, outcomes in each IPI risk group have improved across both limited and advanced disease groups, and smIPI retains utility [11, 12].

The anti-CD20 monoclonal antibody, rituximab, improves survival in limited-stage disease just as it does for advanced-stage DLBCL [13, 14]. The MabThera International Trial (MInT) study found that non-bulky, limited-stage disease treated with R-CHOP had better 6-year event-free survival (EFS) at 84.3% and 6-year OS of 94.9% than CHOP alone. Notably, these survival benefits occurred without a significant increase in toxicity or rate of secondary hematologic malignancy in long-term follow-up [15, 16]. This study helped establish the superiority of chemoimmunotherapy in younger patients with a good prognosis, limited-stage DLBCL.

The treatment of limited-stage DLBCL patients in the modern era has evolved to reconsider the role of RT, the optimal length of systemic therapy, and the emerging role of metabolic imaging via positron emission tomography (PET) in response-adapted management. This review will focus on therapies and studies focusing on limited-stage DLBCL (Table 1), the inherent challenges and future considerations for this disease, and our recommended approach to these patients as diagnostics and therapeutics continue to evolve in the modern era.

**Table 1:** Recent prospective trials and large registry studies in the modern era in limited-stage DLBCL.

	Risk stratification	Study structure	Bulky definition	Radiation dose	EFS	OS	Comments
**Studies in the chemoimmunotherapy and pre-PET era**							
**LYSA/GOELAMS 02-03**	smIPI = 0, 4 cycles of R-CHOP smIPI ≥ 1; 6 cycles of R-CHOP	R-CHOP with RT R-CHOP without RT	>7 cm	40 Gy	5-year: 92% 5-year: 89%	5-year: 96% 5-year: 92%	No statistical significance
**FLYER**		R-CHOP x4 with R-alone x2 R-CHOP x6	>7.5 cm	n/a	3-year: 89% 3-year: 89%	3-year: 99% 3-year: 98%	No statistical significance
**Studies in the chemoimmunotherapy and PET era**							
**S1001**		R-CHOP x4 if negative iPET R-CHOP x3 and IFRT with radioimmunotherapy if positive iPET	>10 cm	36 Gy	5-year: 89% 5-year: 86%	5-year: 91% 5-year: 85%	No statistical significance
**BCCA**	80% had at least one smIPI risk factor	R-CHOP x4 if negative iPET after 3 cycles R-CHOP x3 and IFRT if positive iPET after 3 cycles	n/a	n/a	3-year: 92% 3-year: 60%	3-year OS: 96% 3-year: 83%	Not assessed
**LNH 2009-1B**	Age-adjusted IPI (aaIPI) = 0	R-CHOP x6 R-CHOP x4 if negative iPET after 2 cycles OR R-CHOP x6 if positive iPET after 2 cycles	n/a	n/a	3-year: 89% 3-year: 92%	Not available	No statistical significance

## Is the biology of limited-stage DLBCL distinct from advanced stage?

DLBCL is increasingly understood to be a highly heterogeneous neoplasm with many variations in morphology, gene expression, and biological regulation. The biology of limited-stage DLBCL versus advanced-stage disease is not clearly understood, and it remains to be determined whether it is a manifestation of earlier disease presentation or truly biologically distinct. In advanced-stage disease, known prognostic indicators include cell-of-origin (COO) and deregulation of *MYC* and *BCL2* via rearrangements or protein overexpression. In advanced-stage disease, COO may impact treatment selection [17–20], with GCB cases having favorable prognoses over non-GCB cases [21, 22]. Deregulation of *MYC* and *BCL2* and/or *BCL6* through translocations results in “high-grade B-cell lymphoma with double-hit/triple hit (HGBL-DHL/THL)” and is associated with inferior survival outcomes [23, 24]. Overexpression of the associated proteins, also a negative prognostic indicator in advanced-stage disease, is often dubbed as double-expressor lymphoma (DEL) [25, 26].

Although data regarding COO in limited-stage cases is more scarce, ~60–75% are thought to have GCB origin [27, 28]. The prognostic significance of COO among limited-stage diseases probably parallels advanced-stage disease, although COO breakdown based on stage is scarcely described. In one study, despite similar proportions of limited-stage disease within GCB and non-GCB subtypes, those with non-GCB cell-of-origin fared worse with a 5-year progression-free survival (PFS) of 48% and OS of 56%, compared to 73 and 78% for GCB cases [29], although this was across all stages of DLBCL. In the S1001 trial discussed further below, GCB cases had a favorable PFS over non-GCB subtypes [4].

The prevalence of limited-stage disease in DHL or DEL populations is variable, and also incompletely studied to date. In a study of DHL patients achieving first complete remission, 24% had limited-stage disease [30]; another study of limited-stage disease found only 19% DEL cases and 4% DHL [27]. The impact of DHL or DEL status on prognosis in limited-stage disease has not been directly addressed, but subgroup analyses have found no difference in outcomes for DHL/DEL populations [4, 27, 31].

Another histopathologic variation of DLBCL is a preceding or concurrent indolent disease component. In a recent prospective cohort study of all DLBCL stages, one-third of limited-stage cases had a concurrent follicular lymphoma [32]. COO for these cases of concurrent indolent lymphoma was most often GCB, and outcomes were comparable to *de novo* GCB-DLBCL, across stages. Many clinical trials discussed below exclude cases of transformed indolent lymphoma, or patients who had received prior therapy; as such, the true incidence and significance of transformed indolent disease to limited-stage DLBCL remain unknown.

With much unknown as to the significance of these prognostic biomarkers in limited-stage DLBCL, there is an unmet need and opportunity for future research to better characterize the biology of limited-stage DLBCL, particularly as genomic studies to date [33] have not separated limited-stage from advanced-stage disease – leaving the question of whether limited-stage DLBCL is a biologically distinct entity, entirely unanswered.

## Initial treatment approach: combined modality or chemotherapy alone?

Similar to advanced-stage DLBCL, optimal regimens for limited-stage DLBCL evolved to include rituximab with chemotherapy regimens, followed by the incorporation of PET in initial staging and response assessment. These two landmark changes have influenced contemporary trial design and standard therapies, as the inclusion of RT in treatment of limited-stage DLBCL has continued to be debated. Radiation alone is insufficient as first-line therapy, with high rates of recurrence outside the field of radiation [34, 35], and radiation (when used) is usually part of a combined modality approach. Below we discuss significant studies which address the role of RT in the modern treatment era.

### Studies in the pre-chemoimmunotherapy and pre-PET era

In the pre-rituximab era, a number of studies evaluated combined modality therapy versus chemotherapy alone, with conflicting results. As discussed above, the SWOG 8736 study initially established that three cycles of CHOP plus RT has improved 5-year outcomes [7]. However, the modern application of this trial is limited by a comparator arm that included more chemotherapy cycles than are standard today. In addition, with prolonged follow-up nearing 18 years, there were no statistically significant differences in the PFS or OS of these groups, and there was a notable pattern of continuous relapse in both arms [36]. Two pivotal Groupe d’Etude des Lymphomes de l’Adulte (GELA) trials in the early 1990s suggested excellent outcomes for chemotherapy alone. In the GELA-93-1 study, patients with limited disease and no adverse IPI prognostic factors were randomized to treatment with CHOP and RT or chemotherapy alone with doxorubicin, cyclophosphamide, vincristine, bleomycin, and prednisone (ACVBP) with sequential consolidation. They found improved 5-year EFS and OS for those receiving ACVBP alone than R-CHOP with RT at a dose of 40 Gy [37]. In comparing different chemotherapy regimens, however, the dose intensity of three cycles of ACVBP was estimated to be about 150% of an equivalent three cycles of CHOP. The GELA-93-4 study found no difference in outcomes of patients with limited-stage disease over the age of 60 treated with four cycles of CHOP alone or four cycles of CHOP with RT [38]. There was, however, a heightened risk of secondary malignancy in the radiation arm. An additional trial by the Eastern Cooperative Oncology Group (ECOG) 1484 also showed no benefit for RT in OS after eight cycles of CHOP [6]. As expected, RT provided excellent local control; among refractory patients in this study - only 3 out of 17 patients from the RT arm progressed at original disease sites, compared with 15 out of 31 patients in the chemotherapy-alone arm.

It is important to note that patient populations differed in these trials, which further confounds interpretation. In particular, the inclusion of patients with bulky disease, variable smIPI risk scores, and other features varied, limiting the ability to directly compare the recommendations and conclusions of each study.

### Studies with chemoimmunotherapy in the pre-PET era

The introduction of rituximab improved survival across all stages of DLBCL, including limited-stage disease. Prior to the introduction of PET as a staging and response assessment marker, prospective trials evaluating the role of RT had conflicting results, at least partially based on the inclusion of bulky disease, further examined below.

Building upon S8736, SWOG 0014 added rituximab to the three cycles of CHOP with RT at a dose of 40–46 Gy. The study excluded bulky disease, defined as >10 cm, and required patients to have at least one smIPI adverse risk factor. This phase 2 multicenter study demonstrated a 2-year PFS of 93% and 4-year PFS of 88%; 2-year OS was 95% and 4-year OS was 92%. When compared indirectly to S8736, this was an improvement on both 4-year PFS of 78% and 4-year OS of 88% [39]. Despite including an older population with more adverse risk factors, S0014 suggested improved outcomes with chemoimmunotherapy.

Among patients receiving rituximab along with chemotherapy, what is the impact of consolidative RT? A retrospective study from MD Anderson looked at the impact of RT, and concluded that among all stages of DLBCL treated in the rituximab era (64% of limited-stage patients received 6–8 cycles of R-CHOP), RT improved PFS and OS [40]. Among limited-stage cases, those receiving RT had a 5-year OS of 92% and a 5-year PFS of 82%, compared to 73 and 68%, respectively for those not receiving RT. Notably, however, bulky disease in this study was defined as >5 cm, and a separate analysis of bulky disease with larger size definitions was not conducted.

The prospective study by LYSA and Groupe Ouest-Est des Leucémies et des Autres Maladies du Sang (GOELAMS), LYSA/GOELAMS 02-03, assessed 334 patients with non-bulky limited-stage DLBCL, randomized to R-CHOP with or without RT. Patients with bulky disease defined as >7 cm were excluded, but all smIPI risk scores were included. All patients were randomized to receive RT at a dose of 40 Gy or not, a minimum of four cycles of R-CHOP, and those with smIPI >1 received an additional two cycles of R-CHOP. After four cycles of R-CHOP, all patients had a PET response assessment, and those in a complete remission (CR) continued treatment whereas those with a partial response (PR) received an additional two cycles of R-CHOP regardless of which treatment arm they were enrolled in. There was no statistically significant difference in 5-year EFS at 89% in the R-CHOP alone arm versus 92% in the R-CHOP plus RT arm. Additionally, 5-year OS was not significantly different at 92% for R-CHOP alone versus 96% for R-CHOP plus RT [5]. This trial supports excellent outcomes without RT in non-bulky limited-stage DLBCL, including those with higher smIPI scores.

The UNFOLDER trial was designed as a 2 x 2 trial to assess the benefit of the R-CHOP treatment schedule and inclusion of RT at a dose of 40 Gy. However, as is further discussed in the section dedicated to bulky disease, the trial was stopped early due to excessive events in the arm without RT. There was, however, no difference in PFS or OS between groups receiving RT or not, including when restricted to those with bulky disease [41].

Although the retrospective and smaller studies present contradictory evidence in the rituximab era of the added benefit of RT, the LYSA/GOELAMS 02-03 trial suggests that for patients with non-bulky disease, RT can be omitted without affecting prognosis.

### Studies in the chemoimmunotherapy and PET era

The introduction of PET as a standard imaging modality in lymphoma introduced another variable in management decisions for limited stage DLBCL, with three key areas of impact: staging, end-of-treatment (EOT) assessment for prognosis, and response-adapted investigations. The use of PET for staging often results in “up-staging”, where it is able to detect additional sites of disease in 35% of patients, with 12% resulting in higher staging [42]. One retrospective study showed that EOT scans had a positive predictive value (PPV) of 56% among those with IPI <3, compared with 80% for those with IPI ≥ 3. When used to monitor for relapse, PET has a 95% sensitivity and 97% specificity [43]. Overall, a negative PET scan at EOT portends an excellent prognosis, and surveillance CT imaging beyond 2 years is not recommended [44].

Several prospective trials have evaluated PET-adapted approaches in limited-stage DLBCL, with the goal of limiting both chemotherapy and RT. S1001 is a US Intergroup study prospectively assessing the role of interim PET (iPET) scans after three cycles of R-CHOP in non-bulky disease. Those with a negative iPET, defined as Deauville score three or less, received one additional cycle of R-CHOP whereas those with a positive iPET received 36 Gy of RT followed by ibritumomab tiuxetan radioimmunotherapy with rituximab. One hundred and twenty-eight patients were included in this study, with a median age of 62 years, stage I disease in 62%, 14% with elevated LDH, 66% with head and neck-only involvement, and 43% with extranodal involvement. smIPI was zero in 27%, one in 42%, two in 28%, and three in 4% of included patients. Of these 128 patients, 110 had a negative iPET and did not receive RT. There was no statistically significant difference in the 5-year PFS of 86% for iPET-positive patients and 89% for iPET-negative patients; likewise, there was no statistical difference in the 5-year OS of 85% for iPET-positive patients and 91% for iPET-negative patients. Only six of 132 patients relapsed at a median 4.9 years follow-up time [4]. This PET-adapted approach successfully reduced the number of chemoimmunotherapy cycles and the need for radiation with equivalent and excellent outcomes.

The British Columbia Cancer Agency (BCCA) adopted a PET-based approach where patients achieving a CR by PET after three cycles of R-CHOP were treated with one additional cycle of R-CHOP, while patients with evidence of residual disease—defined by either International Harmonization Project criteria or minimum Deauville score three—also received RT. Eighty percent of the study population had at least one smIPI risk factor, with a 3-year PFS of 92% and 3-year OS of 96% for those with a negative iPET, compared to 60 and 83%, respectively, for those with positive iPET [45, 46]. This regional analysis suggests that a response-based assessment using iPET holds promise in allowing for a reduction in chemoimmunotherapy length and associated toxicity, whereas there remains a need for treatment optimization for patients with positive iPET.

The recent LYSA group study, LNH 2009-1B, further evaluated the role of early PET imaging in assessing whether four cycles of R-CHOP were non-inferior to six cycles. In the experimental arm, those who had a negative iPET after two cycles received a total of four cycles whereas those with a positive iPET after two cycles received a total of six cycles. Early results showed that with a median follow-up of 5 years, the 3-year PFS was 89% in the standard arm where all patients received six cycles of R-CHOP, and 92% in the experimental arm [47]. The non-inferiority of this PET-adapted approach allowing for abbreviated chemoimmunotherapy in those with a negative iPET further suggests that excellent outcomes can be achieved in this population with less treatment.

Similarly, the ongoing OPTIMAL >60 aims to use iPET to evaluate response after two cycles of therapy, with either additional two cycles of therapy for those with a negative iPET or additional cycles with RT for those with a positive iPET. This study also explores the role of other PET-derived biomarkers such as metabolic tumor volume, which have been shown in other studies to be predictive of PFS [48] and OS [49].

## How much chemoimmunotherapy is enough?

Historically, the number of chemotherapy cycles was only abbreviated when adding RT, and studies without RT often delivered more cumulative cytotoxic chemotherapy [7, 36, 37]. However, can chemotherapy cycles be reduced concurrently with omission or reduction of RT? Overall survival in several studies exceeds 90%, raising the possibility of overtreatment for patients at lowest risk.

The randomized FLYER trial addressed whether, among limited-stage DLBCL patients, four cycles of R-CHOP alone were non-inferior to six cycles of R-CHOP. The trial enrolled 588 patients with non-bulky disease and no adverse IPI risk factors, a median age of 48 years, 32% extranodal involvement, and 40% of patients with stage II disease. This study showed that four cycles of R-CHOP were non-inferior to six, with a 3-year PFS of 96% for those who received four cycles, and 94% for six cycles. Likewise, the 3-year OS was 99% for those with four cycles, and 98% for six cycles. Fewer adverse events, including cytopenias and non-hematologic events, occurred in the four-cycle group, as expected with a smaller cumulative dose of chemotherapy [50]. This trial did include two additional rituximab infusions as consolidation, but prior meta-analyses have not shown survival benefit for rituximab consolidation [14, 51, 52]. An added consideration is the ongoing COVID-19 pandemic, where fewer rituximab doses might be advantageous in terms of infectious risk and response to available vaccines; several recent trials suggest that mounting a sufficient immune response is delayed for 12 months after rituximab exposure.

It is important to note that the FLYER trial was limited to patients without risk factors; extrapolating these findings to other groups may not have the same outcomes. Nevertheless, this trial sets a treatment standard for young patients without risk factors and spares higher doses of cumulative chemoimmunotherapy.

## Special management considerations in limited-stage disease

As modern treatment approaches to limited-stage DLBCL have evolved to shorten courses of chemoimmunotherapy and potentially omit RT when incorporating PET-based response assessments, the need to consider special populations that may require modified treatment approaches remains. There is minimal data guiding treatment in the very elderly, but our approach is to select R-CHOP, R-mini-CHOP, or R-CEOP akin to approaches in advanced-stage disease, but limit the number of cycles similar to approaches in limited-stage disease for younger patients. The cases of bulky disease, DHL and DEL, extranodal involvement, CNS prophylaxis, and fully resected disease are discussed below.

### Bulky disease

Bulky disease is variably defined within limited-stage DLBCL; most limited-stage studies exclude stage II bulky disease, and bulky stage I disease is a relatively rare entity. Other studies consider bulky stage II disease akin to advanced stage DLBCL, although cutoffs for defining bulk vary. Studies such as FLYER, LYSA/GOELAMS 02-03, SWOG 0014, and S1001 discussed above all excluded patients with bulky disease and, as such, many of their conclusions must be applied with caution for patients with bulky disease.

The UNFOLDER trial randomized patients with the bulky disease to chemoimmunotherapy with or without RT, where bulky disease was defined as >7.5 cm, and extranodal disease was included. The study was stopped early because of excessive failures based on pre-defined criteria in the non-RT arm among patients with bulky disease [41]. These events were attributed to partial responses requiring localized RT; however, there was no difference in PFS or OS (3-year PFS of 89% vs 81%; 3-year OS of 93% each) between groups receiving RT or not. Although retrospective, a different analysis from MD Anderson included 190 patients who had limited stage disease; 54% received RT, and 48% of those had bulky disease (defined as >5 cm in diameter). They did not find that bulky disease, across all stages, was associated with worse outcomes [40].

Most of these studies have been limited in their ability to definitively address the question of bulky limited-stage disease in part because of sample size of this sub-population. A recent retrospective study in Finland aimed at answering this outstanding question with the adjunctive use of iPET. One hundred twenty-three all-stage DLBCL patients had bulky disease and 44% of these received RT. Among limited-stage patients, the presence of bulky tumor was associated with inferior prognosis, with 2-year PFS of 53% compared to 90% to those with non-bulky disease; however, the benefit of RT in delaying time to progression disappeared after excluding primary refractory cases. They additionally noted that within bulky disease, a negative iPET retained its favorable prognostic benefit, with a 2-year PFS of 87% for those with negative iPET and bulky disease, compared to 57% for those with a positive iPET [53]. While retrospective in nature, this study suggests that RT provides an additional benefit in cases of bulky limited-stage disease such as primary refractory disease, but reinforces that a negative iPET retains its prognostic power even in the presence of other risk factors and that RT may not be necessary.

### Double-hit lymphoma and dual expression of MYC/BCL2

The prognostic significance of DHL and DEL within limited-stage disease is incompletely characterized. One prior retrospective study assessing DHL patients suggested that low-risk DHL patients may benefit from RT in prolonging time to relapse [54]. However, a recent retrospective study [3], focusing on limited-stage *MYC-*rearranged cases showed that, among 104 patients, the overall response rate was 91% to chemoimmunotherapy. The CR rate for those with DHL was 75% compared to 98% for *MYC*-only rearrangements. The 2-year PFS and OS were 78 and 86% for the entire cohort, and did not differ between those receiving RT or among DHL patients. Additionally, for limited-stage patients, there was no demonstrated benefit in using intensive chemoimmunotherapy over R-CHOP for either *MYC-*only rearranged cases or for DHL. Altogether these studies suggest that DHL or DEL cases of limited-stage disease may have a different prognosis than what is expected in advanced-stage disease, although numbers are relatively small. While awaiting further studies to characterize these differences, there is no clear role for intensified treatment for these populations at present.

### Extranodal involvement

Another special population to consider in limited-stage disease is extranodal disease, which has been associated with worse outcomes than nodal disease in retrospective studies in the rituximab era [55], but with conflicting results in the limited-stage population. Approximately 50% of extranodal DLBCL cases present as stage I, [56] posing a unique challenge in management compared to advanced-stage disease. Additionally, the smIPI, unlike IPI, does not consider multiple extranodal sites of disease a risk factor.

Of the studies discussed above, the LYSA/GOELAMS 02-03 study which assessed the role of RT with 4–6 cycles of R-CHOP and included patients with extranodal disease, did not find that the presence of extranodal sites impacted EFS or OS [5]. The S1001 trial similarly did not find worse outcomes for extranodal involvement [4]. Other studies such as FLYER excluded patients with extranodal disease, and MInT allowed clinicians to use RT at their discretion, but did not separately analyze extranodal disease [15, 16].

A recent retrospective study of stage I DLBCL patients [55], however, suggested that extranodal disease is associated with inferior outcomes. Among 341 stage I cases, 66% had extranodal involvement, with the most common sites being bone, stomach, testis, intestine, and breast. 69% of these extranodal patients received RT, compared to 68% without extranodal involvement. Patients with extranodal disease had worse outcomes, with a 10-year OS of 70% and 10-year PFS of 63%, compared to 89 and 85% for nodal disease. Consolidative RT was associated with improved OS and PFS within extranodal disease. Those with a positive EOT PET scan did not have inferior survival, but 75% of these patients received consolidative RT; there was no added benefit from RT for those with a negative EOT PET. All relapses occurred outside the radiation field, and the most common sites were nodal and central nervous system (CNS). Only 8% of patients with extranodal disease relapsed, and of those who did, 30% relapsed at the original disease site. Patients who relapsed after RT all presented with distant sites of relapse. Those with CNS relapse had initial involvement of breast and testes, suggesting that CNS prophylaxis for those with breast involvement may be of benefit. In contrast, there is an ongoing SWOG analysis that shows no adverse impact of extranodal presentation. This retrospective analysis included patients with non-bulky disease from S1001 as well as S0313 and S0014, and found that there was no significant difference in either EFS or OS at 10 years based on whether patients had extranodal or only nodal disease [57].

Another retrospective study of 126 stage I DLBCL patients with extranodal involvement [58] analyzed patterns of relapse, with the most common initial sites including gastrointestinal, bone, and nasopharyngeal disease. Consistent with the above study, they showed a 15% relapse rate, with 79% having distant sites of relapse and 25% being >5 years out from initial diagnosis. Additionally, 32% of relapses involved the CNS, all among cases initially involving the testes (despite prophylaxis), nasopharynx, and nodal disease.

Specifically for limited stage testicular disease, although rare—occurring in ~1% of extranodal cases [59]—a combination of R-CHOP, intrathecal prophylaxis with methotrexate (discussed below), and contralateral testicular radiation of 30Gy in stage I and II patients resulted in a 5-year PFS of 74% and 5-year OS of 85% [60]. This combination of chemoimmunotherapy with radiation and CNS prophylaxis is thus highly effective.

Although further prospective trial data is needed to determine the role of additional therapy for extranodal disease, the inclusion of radiation is controversial. The studies above suggest that by incorporating EOT PET, such response-based assessment may be able to better identify extranodal disease which would benefit from consolidative RT. Additionally, certain presentations of extranodal disease may merit a consideration of CNS prophylaxis, discussed below.

### CNS prophylaxis

There is no data guiding the indication and/or utility of CNS prophylaxis in limited-stage disease. When evaluating the CNS-IPI, features most associated with the risk of CNS relapse that is pertinent in limited-stage disease are extranodal involvement or involvement at a site considered “high risk” such as nasal sinuses [61]. Studies evaluating the CNS relapse risk in limited-stage disease suggest a higher risk of relapse if there was an *MYC* rearrangement; there was no impact of primary disease site [58]. Among relapses, one-third had a CNS relapse. A recent retrospective review of CNS prophylaxis and relapses in all stages of DLBCL found an increased risk of testicular CNS relapse, but no difference in CNS relapse rates between routes of prophylaxis—either systemic high-dose methotrexate (HD-MTX) or intrathecal methotrexate (IT-MTX) [62]. With this lack of evidence of a difference in relapse rates, IT-MTX is often a preferable option for prophylaxis given lower rates of toxicity and delay in systemic therapy.

National Comprehensive Care Network (NCCN) guidelines for CNS prophylaxis, although not specific to limited stage disease, include high-risk disease sites as an indication for prophylaxis, including kidney, adrenal, testicular, and breast [1]. Other retrospective work has suggested that involvement of sites such as paranasal sinuses, breasts, and testes should also receive CNS prophylaxis [63, 64]. Overall, there is insufficient data to definitively guide decision-making regarding CNS prophylaxis, and more data is needed.

### Fully resected disease

In some cases of limited-stage disease, the entirety of the tumor may be resected during the diagnostic biopsy, or in other special cases such as obstruction from intestinal extranodal involvement [65]. Traditionally, these patients were given the same duration of therapy as other limited-stage disease patients. Subgroup analyses of existing studies have attempted to better answer whether fully resected disease allows for abbreviated therapy, although this varies by initial site of disease and other considerations.

A phase 2 study conducted by the Consortium for Improving Survival of Lymphoma (CISL), CISL 12-09, set out to determine the safety and efficacy of an abbreviated three cycles of R-CHOP after total resection of limited stage disease. At a median follow-up time of 39.5 months, only one patient out of 22 progressed, with an estimated 2-year OS was 95% [66]. In extended follow-up at 5 years, no additional events related to disease progression or death occurred [67]. Although limited by study size, this supports a limited systemic treatment course for patients with fully resected limited stage DLBCL.

Analysis of patients enrolled in the Positron Emission Tomography-guided Therapy of Aggressive non-Hodgkin Lymphomas (PETAL) trial [68] found that 52 patients had fully resected stage I disease by baseline PET, with most patients receiving 6 cycles of R-CHOP. Those with surgically resected disease under age 60 had an improved 2-year PFS and OS of 100% compared to 92 and 95%, respectively, for those with incompletely resected disease; there were no statistically significant differences between groups for those over age 60 [65]. Furthermore, given the results of the FLYER trial above, it is reasonable to extrapolate that four cycles of chemoimmunotherapy would be sufficient.

A retrospective study of 250 primarily intestinal, limited-stage patients receiving six cycles of CHOP or R-CHOP showed a significantly better CR rate in the combined surgery and chemotherapy group of 85% compared to 64% for those receiving chemotherapy alone, who also had local relapses more frequently. Of those who underwent surgery, 60% were for mass resection and 31% for obstruction. Surgically resected patients had a 3-year PFS of 82% and a 3-year OS of 91%, compared to 52 and 62% for chemotherapy alone. There was no survival benefit, however, in surgical resection of advanced-stage cases involving intestinal disease [69]. Importantly, this finding suggests that while in advanced-stage disease surgical resection does not provide a survival benefit, in specific anatomical considerations of limited-stage disease, surgical resection may be useful.

## Our approach to management

**Fig. 1:**
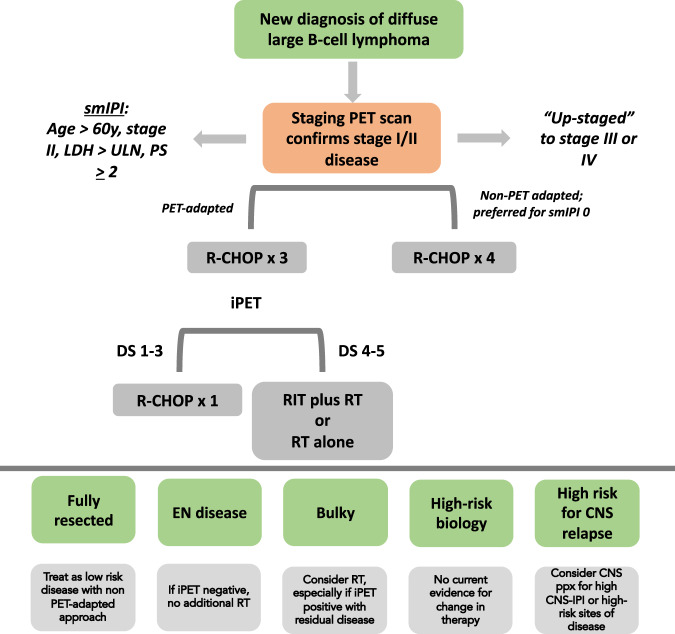
Our approach to the management of limited stage diffuse large B-cell lymphoma (DLBCL). Abbreviations: iPET interim PET, smIPI stage modified IPI, RIT radioimmunotherapy, DS Deauville score, ppx prophylaxis, PS performance status, ULN upper limit normal.

The current outcomes for patients with limited stage DLBCL are outstanding, and the field has evolved to limit both short- and long-term toxicities without compromising efficacy. Our approach (Fig. 1) is based on initial clinicopathologic features, incorporation of the smIPI, and the results of interim PET scanning. Patients with a low smIPI or negative iPET, including fully resected disease, can be treated with a maximum of four cycles of R-CHOP without consolidative radiotherapy. For those with a positive iPET, additional radiation and/or additional chemoimmunotherapy may hold a survival benefit. RT is also a consideration for those with bulky (>7.5 cm) or extranodal disease, with additional guidance for individual cases as discussed above. Such an approach minimizes the toxicity of therapy without sacrificing benefit of outcomes. Additional factors also need to be considered, such as CNS prophylaxis for high-risk disease sites such as testicular, breast, and nasopharyngeal involvement, and limited treatment courses for those with the fully resected disease at staging.

Upcoming studies in limited stage DLBCL will aim to further refine the role of earlier iPET in reducing the need for further therapy. Additionally, further insight into the specific disease biology of limited DLBCL should be pursued to further our understanding of new DLBCL classification that may not be dependent on stage alone. This may change the diagnosis and management of limited-stage DLBCL and has the potential to even further improve outcomes and toxicities in a disease that has seen great strides into the modern treatment era.
